# Age group prediction with panoramic radiomorphometric parameters using machine learning algorithms

**DOI:** 10.1038/s41598-022-15691-9

**Published:** 2022-07-09

**Authors:** Yeon-Hee Lee, Jong Hyun Won, Q.-Schick Auh, Yung-Kyun Noh

**Affiliations:** 1 Department of Orofacial Pain and Oral Medicine, Kyung Hee University Dental Hospital, Kyung Hee University, #26 Kyunghee-daero, Dongdaemun-gu, Seoul, 02447 Korea; 2 Department of Computer Science, Hanyang University, Seoul, Korea; 3 School of Computational Sciences, Korea Institute for Advanced Study (KIAS), Seoul, 02455 Korea

**Keywords:** Anatomy, Biomarkers, Health care, Medical research

## Abstract

The aim of this study is to investigate the relationship of 18 radiomorphometric parameters of panoramic radiographs based on age, and to estimate the age group of people with permanent dentition in a non-invasive, comprehensive, and accurate manner using five machine learning algorithms. For the study population (209 men and 262 women; mean age, 32.12 ± 18.71 years), 471 digital panoramic radiographs of Korean individuals were applied. The participants were divided into three groups (with a 20-year age gap) and six groups (with a 10-year age gap), and each age group was estimated using the following five machine learning models: a linear discriminant analysis, logistic regression, kernelized support vector machines, multilayer perceptron, and extreme gradient boosting. Finally, a Fisher discriminant analysis was used to visualize the data configuration. In the prediction of the three age-group classification, the areas under the curve (AUCs) obtained for classifying young ages (10–19 years) ranged from 0.85 to 0.88 for five different machine learning models. The AUC values of the older age group (50–69 years) ranged from 0.82 to 0.88, and those of adults (20–49 years) were approximately 0.73. In the six age-group classification, the best scores were also found in age groups 1 (10–19 years) and 6 (60–69 years), with mean AUCs ranging from 0.85 to 0.87 and 80 to 0.90, respectively. A feature analysis based on LDA weights showed that the L-Pulp Area was important for discriminating young ages (10–49 years), and L-Crown, U-Crown, L-Implant, U-Implant, and Periodontitis were used as predictors for discriminating older ages (50–69 years). We established acceptable linear and nonlinear machine learning models for a dental age group estimation using multiple maxillary and mandibular radiomorphometric parameters. Since certain radiomorphological characteristics of young and the elderly were linearly related to age, young and old groups could be easily distinguished from other age groups with automated machine learning models.

## Introduction

Age estimation is an important step in biological identification in the forensic field. An age group estimation is necessary to identify the dead, and is crucial in living individuals to clarify various legal queries and solve civil or judicial problems^1^. Several methods using different body parts have been proposed for an age estimation^2^. However, a dental age estimation using radiographic tooth development and a tooth eruption sequence has been found to be more accurate than other methods^3^. Tooth and dental tissue are mainly genetically developed and are less likely to be affected by nutritional and environmental factors, and there is little deformation owing to external chemical and physical damage^4^.

The most common method for dental age estimation is Demirjian’s method, which estimates chronological age by using eight theoretically defined stages of tooth development^5^. Although this method is a straightforward scoring process, it requires human labor, and its subjects are usually limited to children and adolescents. Although a few modified versions of Demirjian’s method have been proposed^6,7^, they all share fundamental limitations of limited applicability to young subjects and unavoidable human judgement, which possibly give high bias to the estimation. In addition, Gustafson's Method is widely accepted^8^; however, only an evaluation of the ground sections of the teeth is possible, and thus there is an evident limit in that a tooth must be extracted both post- and ante-mortem.

Compared to children and adolescents, estimating the ages of living or deceased adults using their permanent dentition is extremely challenging. However, some dental factors are known to be associated with an increase in age, which can provide an important clue for estimating the ages of adults. Tooth surface loss is a macroscopically irreversible process that accumulates with age^9^. It has been reported that the pulpal area of maxillary and mandibular molars decreases with age^10,11^. In addition, the extent and severity of dental caries increases with age, causing tooth damage, dental treatment, and/or tooth loss. In the elderly, implant placement in the edentulous area has become common, and the peak age for implant placement is between 60 and 75 years of age^12^. Periodontitis occurs in all age groups but tends to increase with age^13^. However, aging does not cause dental tissue damage or periodontitis, and it is necessary to collectively examine how various dental radiomorphometric parameters are related to aging.

Machine learning is a field of artificial intelligence that has become important in the areas of forensic medicine and dentistry. Machine learning algorithms that are strong in pattern and rule recognition are suitable for automatic dental age estimation^14^. In addition, machine learning algorithms learn a prediction function directly from data without any human intervention, which effectively removes error from human labor as well as the need to construct a prediction function from scratch^15^. A few studies have recently attempted to overcome the shortcomings of conventional methods by using machine learning algorithms with panoramic radiographs (PR), including an end-to-end system based on a convolutional neural network (CNN) model with raw PR^16^ and the proposed modified conventional method aided with machine learning algorithms^17^.

In the present study, we aimed at providing a dental age-group estimation model based on machine learning using 18 radiomorphometric parameters derived from PRs. As our hypothesis, which we will prove through machine learning, (1) the parameters that mainly contribute to age estimation may differ depending on age, (2) linear models can extract age-related information without the power of nonlinearity and thus can achieve quite a good prediction performance, and (3) the prediction accuracy of machine learning algorithms will be higher than that of traditional age estimation methods. Thus, we conducted binary classifications for each age group using five machine learning models, i.e., two linear and three nonlinear models, and analyzed the learned feature weights of the former. We investigated the configuration by learning the subspace using a Fisher discriminant analysis (FDA). To the best of our knowledge, this is the first study on dental age-group estimation using five linear and non-linear machine learning algorithms with various PR-derived radiomorphometric parameters.

## Materials and methods

The research protocol for this study was reviewed in compliance with the Helsinki Declaration and was approved by the Institutional Review Board of the Kyung Hee University Dental Hospital in Seoul, South Korea (KHD IRB). Informed consent was obtained from all participants. In patients under the age of 18 years, informed consent was obtained from a parent and/or legal guardian.

**Figure 1:**
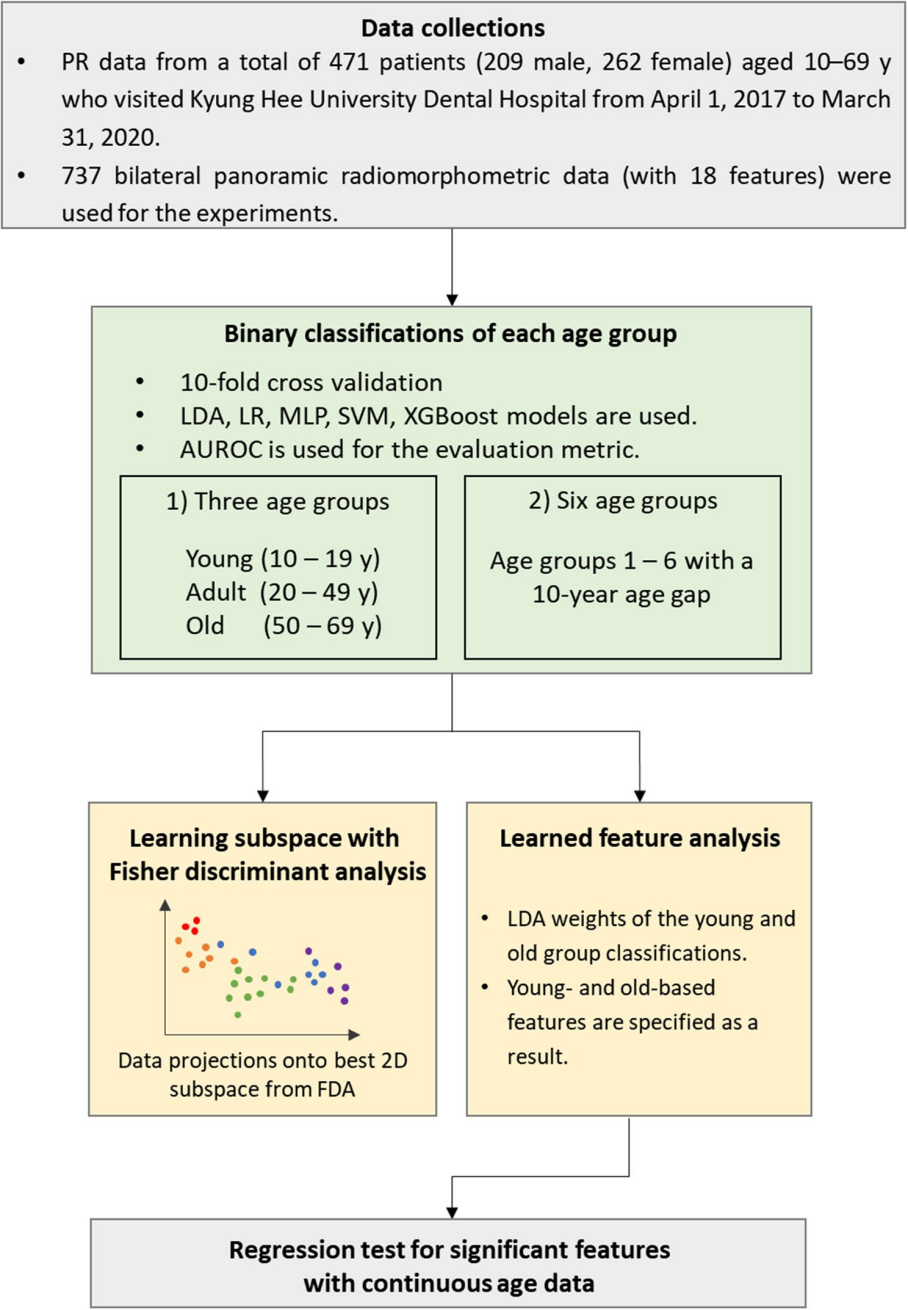
Flow chart of the present study. *LDA* linear discriminant analysis, *LR* logistic regression, *SVM* kernelized support vector machines, *MLP* multilayer perceptron, *XGBoost* extreme gradient boosting. Lastly, Fisher discriminant analysis (FDA) was used to visualize the data configuration.

The overall flow chart of this study is shown in Fig. 1. The study sample consisted of 471 healthy patients (209 men and 262 women) with a known age of 11–69 y (mean age of 32.57 ± 17.81 years). All participants had PRs and were receiving dental care at Kyung Hee University Dental Hospital between April 1, 2017 and March 31, 2020. Multiple anatomical parameters of the maxilla and mandible that are expected to change with increasing age were collected from the PRs of the patients.

The inclusion criteria for patient selection were as follows: (1) all parameters clearly visible in the PR image, (2) permanent dentition, (3) full eruption of the selected maxillary canines and four first molars into the oral cavity in all quadrants, and (4) roots of selected canines and molars fully formed. None of the subjects had any developmental, endocrine, or nutritional disorders, and none had any special dental pathologies, such as amelogenesis imperfecta or dentinogenesis imperfecta. Patients with systemic disorders that could affect tooth maturation, eruption, and bone growth were excluded from the study, as were any teeth with developmental anomalies. Patients with a history of maxillofacial, maxillary, or mandibular surgery were also excluded, as were those with primary or mixed dentition.

PRs were acquired using an X-ray imaging machine (Promax 2D; Planmeca Oy, Helsinki, Finland), with the same distance between the film and the X-ray tube, beam angulation, film size, and exposure time used in all patients. The head position of the patients was maintained using a chin rest and bite guide. The optimal image density and contrast were achieved at 16-s exposure settings of 84 kVp and 16 mA, with a magnification factor of 1.20. PR data were saved in Digital Imaging and Communications in Medicine (DICOM) files, whereas the Picture Archiving Communication System (PACS) was used to analyze the DICOM data. All measurements were conducted using the utility tool in the tool bar of the PACS program, and each factor was measured bilaterally. The length was measured in millimeters and the area was measured in square centimeters. All measurement procedures and investigations were conducted by two investigators (YHL and QSA), and the internal consistency was expressed as a Cronbach’s α value of 0.9 or higher (p < 0.001).

For the age-group estimation, we first divided the patients into three groups: young (10–19 years), adult (20–49 years), and old (50–69 years). To test the estimation for more specific ages, we also used the following six age groups, each representing a 10-year span: age group 1 (10–19 years), age group 2 (20–20 years), age group 3 (30–39 years), age group 4 (40–49 years), age group 5 (50–59 years), and age group 6 (60–69 years). For training, we used 90% of the dataset as a training set, and the remaining 10% was used as a test dataset. Because our data were collected from teeth on both sides, we enforced bilateral data from the same individual to be in the same training or test set because the learning algorithms would otherwise test data that are quite similar to those in the training set.

### Selected features on panoramic radiography

We investigated 18 parameters (Table 1) of the maxilla and mandible, including quantitative measurements of the maxillary canines, maxillary and mandibular first molars, and the vertical positions of the MC and mental foramen (MF). All such features are expected to be related to chronological age^11^.

**Table 1:** Eighteen selected features in panoramic radiograph and their brief descriptions.

Number	Feature	Description
1	MC to AC	Distance between the mandibular canal and alveolar crest
2	U-Tooth area	Tooth area of the upper first molar
3	U-Pulp area	Pulp area of the upper first molar
4	L-Tooth area	Tooth area of the lower first molar
5	L-Pulp area	Pulp area of the lower first molar
6	U-Root length	Root length of the upper canine
7	Teeth	Total number of teeth
8	U-Endo	Number of endodontic treatments in lower dentition
9	L-Endo	Number of endodontic treatments in upper dentition
10	U-Crown length	Crown length of the upper canine
11	Root to IAN	Distance from first molar root tip to inferior alveolar nerve
12	MF to MB	Distance from mental foramen to mandibular border
13	MF to AC	Distance from mental foramen to alveolar crest
14	U-Crown	Number of crown treatments in upper teeth
15	L-Crown	Number of crown treatments in lower teeth
16	U-Implant	Number of implants in upper teeth
17	L-Implant	Number of implants in lower teeth
18	Periodontitis	Presence of periodontitis

1. Tooth length and shape
  To determine the length of the maxillary canine, the crown and root lengths (U-Crown Length and U-Root Length) were measured. To identify the pulp and tooth areas of the upper and lower first molars, the tooth (U-Tooth Area and L-Tooth Area) and pulp area (U-Pulp Area and L-Pulp Area), and the maxillary and mandibular first molars were investigated.
2. Position of mandibular canal
  At the position of the mandibular first molar, a tangent line was drawn to the superior border of the mandibular canal (MC). A vertical line was drawn from the tangent line to the alveolar crest (AC) of the mandibular first molar. Next, the distance between the MC and AC (MC to AC) was measured, and the relative vertical position of the MC in the mandible was investigated.
3. Position of mental foramen
  At the position of the mental foramen (MF), a tangent line was drawn to the inferior cortical outline of the mandible, and a vertical line was drawn in the direction of the AC. The distance from the mental foramen to the mandibular border (MF to MB) and the distance from the mental foramen to the alveolar crest (MF to AC) were measured. Thus, the relative vertical position of the MF according to the increase in age was investigated.
4. Tooth and pulp area of first molar
  The whole tooth (U-Tooth Area and L-Tooth Area) and pulp area (U-Pulp Area and L-Pulp Area) of the four maxillary and mandibular first molars were measured in the PR image of each individual. We investigated the changes in the whole tooth and the pulp areas with increasing age.
5. Number of treated teeth and total number of teeth
  We conducted a visual assessment of the number of endodontically treated teeth, full veneer crowns, and implant prostheses in the upper and lower dentition. The total number of teeth were also calculated. This process was conducted by two investigators (YHL and QSA). A total of 28 teeth (seven per quadrant) were set as the normal number from the incisors to the second molars. The third molars were excluded from the evaluation.
6. Presence of periodontitis
  Based on the distance from the alveolar bone level to the cemento-enamel junction, when alveolar bone destruction was observed at ≥ 30% of the probing sites, we diagnosed the condition as periodontitis. To test the repeatability of the measurements, 30 patients were randomly re-evaluated 2 weeks after the initial measurements. The test–retest reliability for these analyses, represented using an inter-class correlation (ICC), ranged from 0.91 to 0.99, indicating an excellent reliability.

### Age group determination using linear and nonlinear machine learning models

Binary classifications for each age group from other age groups were conducted to investigate how well each age group could be separated from the others. We first conduct the classification for three age groups (young, adult, and old) and continued to the six age groups. The main purpose of the classifications was to measure how well different ages could be discriminated, with the subsidiary purpose to validate the hypothesis that linear models can perform sufficiently well in comparison to more flexible nonlinear models based on the expectation that age-related information can be easily extracted without applying nonlinearity. Considering this, we used both linear and nonlinear machine-learning models and tested their AUCs for each classification. As the rule of thumb for interpreting the AUC value, AUC = 0.5 (no discrimination), 0.6 ≥ AUC > 0.5 (poor discrimination), 0.7 ≥ AUC > 0.6 (acceptable discrimination), 0.8 ≥ AUC > 0.7 (excellent discrimination), and AUC > 0.9 (outstanding discrimination)^18^. Even though the present study mainly interested in each group’s separability from others using a variety of machine learning models, multi-label classifications also performed using LDA and XGBoost models to show how accurate the models could be in the multi-label classification problem and to find out whether the nonlinear model can outperform the linear model.

The description and hyperparameter settings for the machine learning models we used are as follows: A logistic regression (LR) and linear discriminant analysis (LDA) were used for the linear models. Although they both use the linear discriminating function $\mathrm{f}\left({w}^{T}x + b\right)$ for classification with parameters $\mathrm{w} \mathrm{and} b$, they learn them quite differently. LR learns them as parameters maximizing a posterior represented by sigmoid functions, whereas LDA learns them by assuming that the given data of each class $\mathrm{c}$ follow a Gaussian density function, whose mean is ${\upmu }_{c}$ and the covariance is $S$. Here, ${\upmu }_{c}$ is the mean of the data with class $c$, and $S$ is the covariance of the whole data regardless of age group. The optimal parameter $w$ was then obtained using ${S}^{-1}\left({\upmu }_{1} -{\upmu }_{0}\right).$ Because our dataset is class-imbalanced and fairly small, we regularized the covariance $\mathrm{S}$ by setting $\mathrm{S}=\mathrm{S}+\uplambda I$ with a small $\mathrm{\lambda of}>0$.

Next, for the nonlinear models, support vector machines (SVM), multilayer perceptron (MLP), and a gradient boosting ensemble model (XGBoost) were used. Originally, the SVM is a linear model using a large margin obtained from a convex optimization, although its main strength comes from applying nonlinear kernels, which enable the model to classify nonlinear data^19^. Gaussian kernels were used in our experiments. MLP is a neural network algorithm that can classify nonlinear data using multiple layers equipped with nonlinear activation functions. Because it often suffers from an overfitting when training using small samples, we set the model to have only one hidden layer with two logits with a sigmoid activation. For the optimization, we used a limited memory version of the Broyden–Fletcher–Goldfarb–Shanno solver method with a fixed learning rate of ${10}^{-5}$ and updated the parameters 10 times. In the prediction of the age group classification, the area under the receiver operating characteristic curves for classifying individual age groups was used in five different machine learning models.

Finally, we selected the gradient boosting ensemble model called XGBoost. This model is trained with an ensemble of hundreds of decision trees with various regularization techniques, such as the number of leaves, shrinkage, and randomized tree subsampling. Because all of these are solely for the higher prediction performance, we expected this model to outperform the others, giving us a benchmark score.

### Learning the subspace using a Fisher discriminant analysis

To explain the classification results and show that our data indeed embed the chronological age, we obtained the subspace using a Fisher discriminant analysis (FDA) with six age grouped data. The FDA captures $k$ vectors ${w}_{1},{w}_{2},\dots ,{w}_{k}$ maximizing the separation between centroids (e.g., means) of all age groups (${S}_{B}$) and minimizing the covariance within groups (${S}_{W}$) at the same time:

$${\mathrm{max}}_{W}\frac{tr\left({W}^{T}{S}_{B}W\right)}{tr\left({W}^{T}{S}_{W}W\right)},$$

with $\mathrm{W} = \left[{w}_{1},{w}_{2},\dots ,{w}_{k}\right]\in {R}^{D\times K}$. The optimal solution for this maximization problem is equivalent to the eigenvectors of ${S}_{W}^{-1}{S}_{B}$. Here, the number of eigenvectors to choose is limited to the number of groups minus one (i.e., $k = 5$ with six age groups). For visualization, we selected the top-two eigenvectors with the largest eigenvalues, and then projected the data and means of the groups onto the subspace spanned by these two eigenvectors.

### Analyzing the learned feature weights of linear discriminant analysis classifiers

An additional feature analysis was conducted by analyzing the learned features of LDAs to understand the features that are important for classifying specific age groups. Specifically, we aimed to identify young and old-specific features. For this, we first compared the learned feature weights of LDAs from the young (age 10–19 years versus 20–69 years) and old group (age 10–49 years versus 50–69 years) classifications. We then selected the features whose weights were in the same direction under both classifications as age-specific features. To ensure that our selections are indeed age-related, we constructed a linear regression model and tested its prediction using true age data. The normalized mean squared error (NMSE) was used as the prediction score. To test the statistical significance of individual features, we also conducted two-tailed t-tests for each feature ${D}_{i}$ with hypotheses ${H}_{0}:{D}_{i}=0$ versus ${\mathrm{H}}_{1}:{\mathrm{D}}_{\mathrm{i}}\ne 0$ in the regression model, which indicates the importance of each feature for the regression. Furthermore, to specify feature importance of not only the linear model but also a nonlinear one, SHAP values were calculated and analyzed from the trained XGBoost.

### Statistical analysis

Descriptive statistics are reported as the mean ± standard deviation or numbers with percentages, where appropriate. Three different statistical software programs, i.e., IBM SPSS Statistics (Version 22.0; IBM, Armonk, NY, USA), R (Version 4.0.2; R Foundation for Statistical Computing, Vienna, Austria), and Python (Version 3.7.4; Python Software Foundation, DE, USA) were used for all statistical analyses. A t-test was conducted to determine whether there was a statistical difference in the AUC values of each machine learning model and whether there was a gender difference in the values. Analysis of variance (ANOVA) with a post-hoc analysis was used to determine whether there was a statistical difference between the AUC values of linear models (LDR and LR) and nonlinear models (XGBoost). After specifying young- and old-specific features, their predictive power was obtained using a simple linear regression. Statistical significance was set at a two-tailed p-value of < 0.05. All measurements and investigations were conducted by two investigators. Internal consistency was represented using Cronbach’s α, and test–retest reliability was represented using ICC.

### Consent for publication

The authors declare no potential conflicts of interest with respect to the research, authorship, or publication of this article.

## Results

### Classification result of each age group with linear and nonlinear models

**Figure 2:**
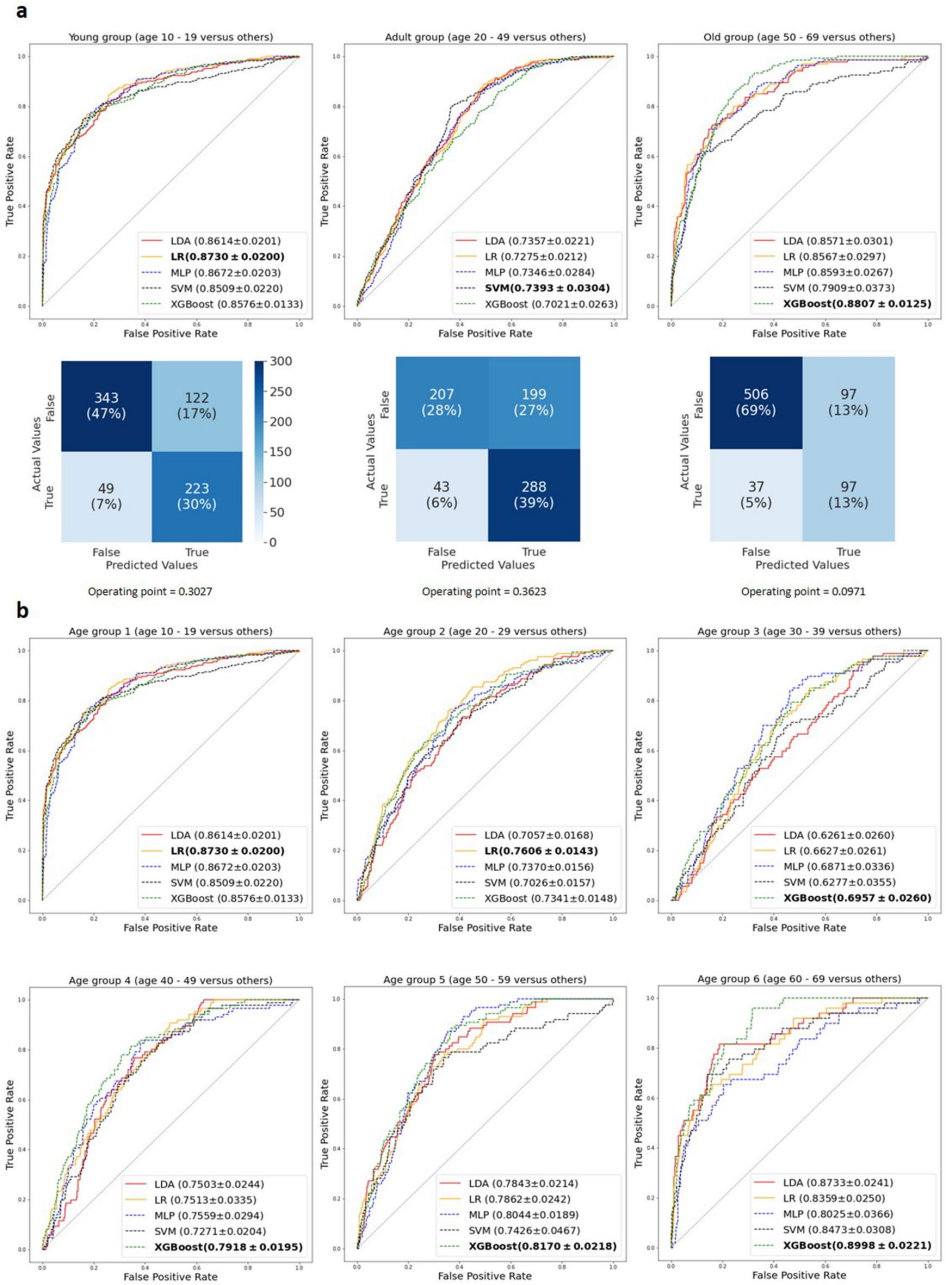
Prediction results of each age groups. ROC curves with the mean AUCs and standard errors of the folds of the five models and confusion matrices in the three age-group classifications (**a**) and ROC curves of six age-groups case (**b**). The bold text in each curve plot represents the model with the best score (mean AUC).

Figure 2 shows the classification results of three age groups (young, adult, and old groups) with each group’s receiver operating characteristic (ROC) curve and confusion matrix (a), and the AUC curves of six age groups (age group 1–6 based on a 10-year age gap) (b). In each curve plot, we presented the mean AUC and standard error obtained from each model in ten folds. The curve was obtained by concatenating the logits of all ten folds and then calculating the true positive (TP) and false positive (FP) rates using some fixed thresholds. Thus, it indicates the overall prediction performance of the entire dataset from the specific model. Bold text for each age group indicates the best machine learning model with the highest mean AUC value. The confusion matrix of each age group was obtained from the logits of LDAs in all 10 folds (i.e., the logits appended from each fold, which includes 10% of whole data set). Thus, overall prediction results were obtained from the ROC curves at optimal operating points.

In the three age groups, the overall best AUCs were achieved in the young group (10–19 years) where their mean AUC scores ranged from 0.8509 to 0.8730, indicating an excellent discrimination. Regardless of the type of machine learning model in the young group, the mean AUC value was 0.85. The next highest AUC scores were observed in the old group (50–69 years), and the AUC scores ranged from 0.7909 to 0.8807. The middle age group, i.e., the adult group (20–49 years), showed much lower mean AUCs than other two groups, with scores of approximately 0.73 s, which demonstrates an acceptable discrimination.

In the follow-up six age group classifications, the best scores were found in age groups 1 and 6, with the mean AUCs ranging from 0.8509 to 0.8730 and 8025 to 0.8998, respectively. The lowest AUC scores were obtained in age group 3 (30–39 years) with AUCs of approximately 0.7 s. Taken together with the scores of other age groups, we can see here that the prediction performances were the highest in the two extreme age groups (the youngest and oldest groups) and worsened in the middle-aged groups.

For both the three and six age groupings, the prediction performances of the linear models were not significantly different from the nonlinear models. Compared to XGBoost, which was used as a representative non-linear model, the AUC values of the linear models (LDA and LR) did not differ statistically (p > 0.05) under all classifications (Table 2). As expected, the linear model could extract as much discriminant information as the nonlinear model, and the accuracy of the prediction was acceptable.

**Table 2:** Age group differences in the AUC values of LDA, LR, and XGBoost.

Machine learning model	Linear model		Nonlinear model	p-value
	LDA	LR	XGBoost	
**Three age groups**				
Young (10–19 years)	0.8614 ± 0.0201	0.873 ± 0.0200	0.8676 ± 0.0133	0.883
Adult (20–49 years)	0.7357 ± 0.0221	0.7275 ± 0.0212	0.7021 ± 0.0263	0.485
Old (50–69 years)	0.8571 ± 0.0301	0.8567 ± 0.0297	0.8807 ± 0.0125	0.505
**Six age groups**				
Group 1 (10–19 years)	0.8614 ± 0.0201	0.8730 ± 0.0200	0.8576 ± 0.0133	0.883
Group 2 (20–29 years)	0.7057 ± 0.0168	0.7606 ± 0.0143	0.7341 ± 0.0148	0.245
Group 3 (30–39 years)	0.6261 ± 0.0260	0.6627 ± 0.0261	0.6957 ± 0.0260	0.407
Group 4 (40–49 years)	0.7503 ± 0.0244	0.7513 ± 0.0335	0.7918 ± 0.0195	0.338
Group 5 (50–59 years)	0.7843 ± 0.0214	0.7862 ± 0.0242	0.8170 ± 0.0218	0.382
Group 6 (60–69 years)	0.8733 ± 0.0241	0.8359 ± 0.0250	0.8998 ± 0.0221	0.453

The AUC values of each machine learning algorithm were obtained, and an ANOVA with a post-hoc analysis was used to determine whether there was a gender difference in these values. P-values of less than 0.05 (*) were considered statistically significant. *LDA* linear discriminant analysis, *LR* logistic regression, *XGBoost* extreme gradient boosting.

### Multi-label classification using linear and nonlinear models

**Figure 3:**
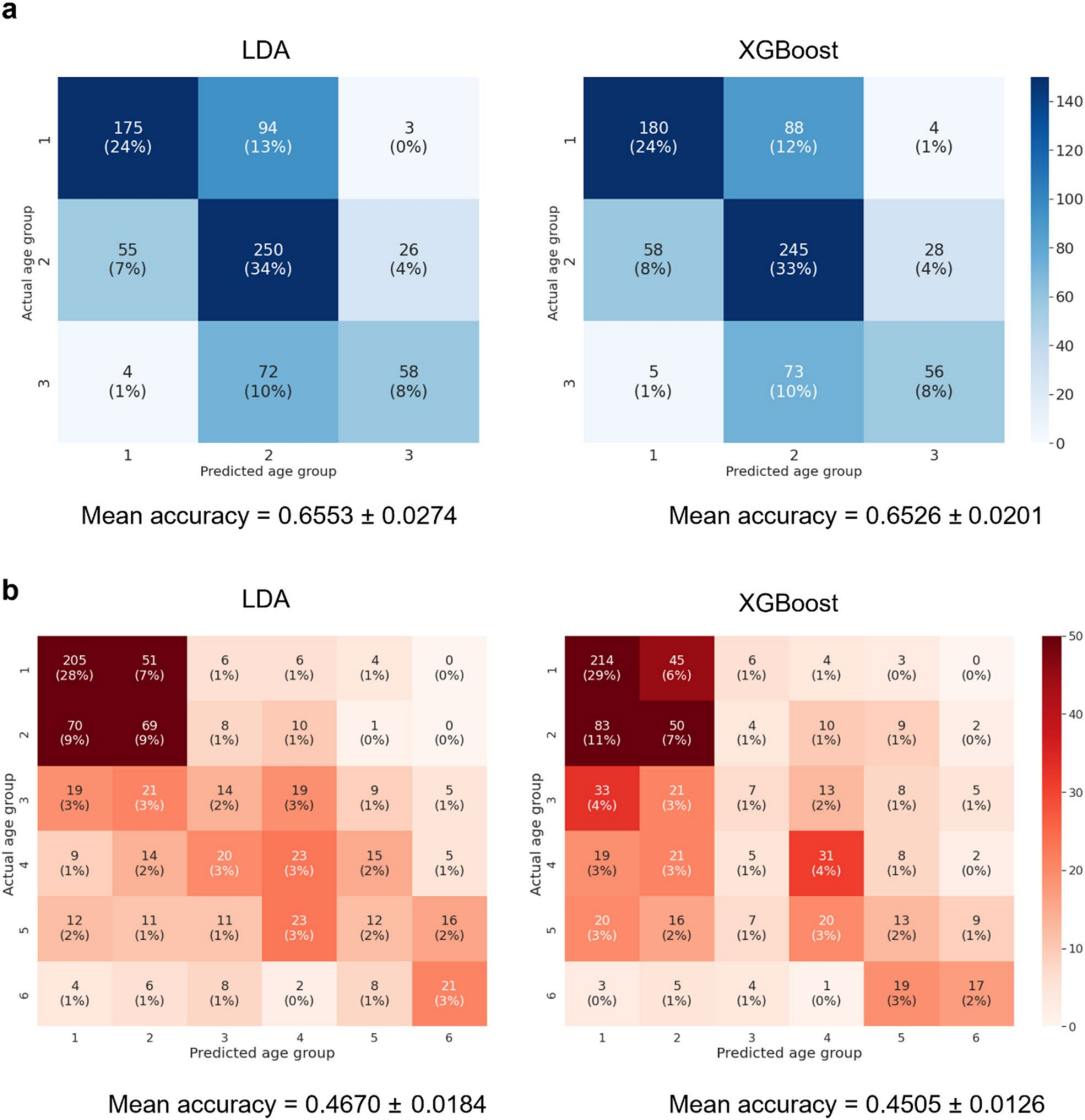
Multi-label classification results using LDA and XGBoost.

Additional experiments with multi-label classification problems were conducted to investigate the prediction accuracies of machine learning models and whether a nonlinear model can outperform a linear model. The results are shown in Fig. 3. In the three age groups prediction, the mean accuracy of LDA across all ten folds was 0.6553 ± 0.0274 and XGBoost was 0.6526 ± 0.0201. In the six age groups case, the prediction accuracies were dropped to 0.4670 ± 0.0184 for LDA and 0.4505 ± 0.0126 for XGBoost. Whether age was divided into three or six groups, the two models showed not significantly different predictive performance. Both age group models showed biased predictive accuracy for younger age groups, especially in the six age group models.

### Gender differences in the AUC values of linear and nonlinear models

Table 3 shows gender differences in the AUC values of the machine learning models. Similar to when analyzing the entire data, the young group had the largest AUC value, followed by the old group, and the adult group had the smallest prediction accuracy value among the three age groups for both sexes. This trend was also observed when the machine learning model was applied by dividing between male and female. The mean scores for the AUC were higher for males than for females in all classifications; however, there was only a significant difference in the young group with XGBoost (males versus females, 0.8668 ± 0.0208, p-value of 0.0297). When the machine learning model was applied to the three age groups by dividing the data by gender, LR for both males and females had the best prediction accuracy, whereas for the old group, LDA for males and LR for females were more accurate.

**Table 3:** Gender differences in the AUC values of LDA, LR, and XGBoost.

Three age groups	Machine learning models	Male	Female	p-value
		(n = 303)	(n = 434)	
Young group	LDA	0.8721 ± 0.0290	0.8227 ± 0.0278	0.2587
	LR	**0.8939 ± 0.0249**	**0.8371 ± 0.0219**	0.1220
	XGBoost	0.8668 ± 0.0208	0.7819 ± 0.0268	0.0297*
Adult group	LDA	0.6535 ± 0.0390	0.6260 ± 0.0461	0.6712
	LR	**0.7092 ± 0.0245**	**0.6919 ± 0.0300**	0.6757
	XGBoost	0.6840 ± 0.0280	0.6214 ± 0.0225	0.1160
Old group	LDA	**0.8735 ± 0.0233**	0.8372 ± 0.0452	0.5097
	LR	0.8454 ± 0.0257	**0.8429 ± 0.0392**	0.9613
	XGBoost	0.8088 ± 0.0327	0.8131 ± 0.0338	0.9307

The results of the gender comparison were obtained using t-tests. Statistical significance was set at P < 0.05. *LDA* linear discriminant analysis, *LR* logistic regression, *XGBoost* extreme gradient boosting. The model with the highest AUC value shown in bold in each group.

### Learned subspace from FDA reveals the chronological order of age groups

**Figure 4:**
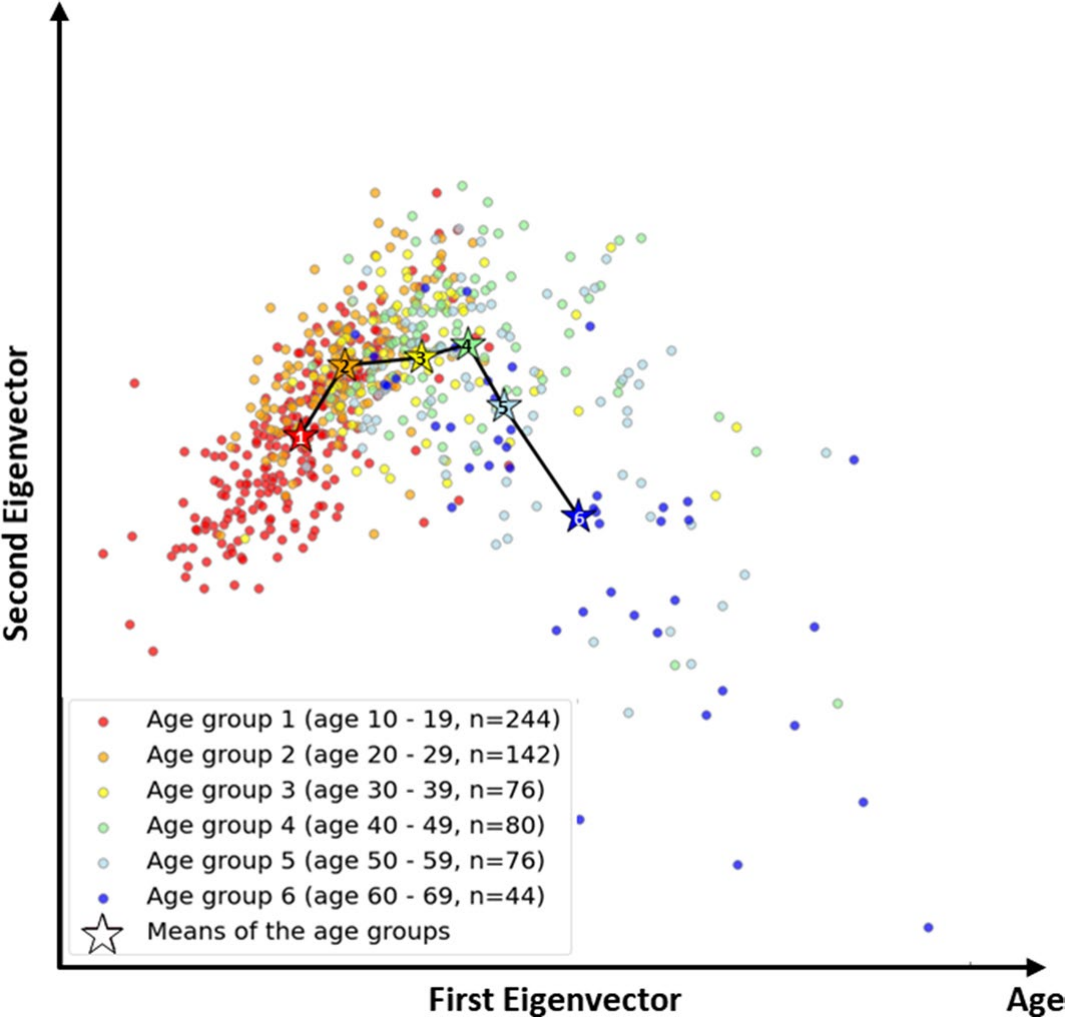
Original 18-dimensional data projections mapped into the 2D space learned from Fisher discriminant analysis. Horizontal and vertical axes are the first and second eigenvectors, where the horizontal axis represents age. The black line concatenating the means of the six groups clearly reveals their chronological order, which supports our data, i.e., embedded age information. The means of the groups are denoted as stars with the group numbers.

Figure 4 shows the result of projecting 18-dimensional data into a 2D space obtained from the FDA. The subspace here is composed of two eigenvectors (each axis) separating out the six age groups as much as possible. By concatenating, the mean of each age group was indicated by the star, and it was confirmed that the chronological order of age groups 1–6 was ideally arranged. This implies that the data we use embed the chronological age, which is easily captured in a linear space. This visualization confirms our hypothesis that the given 18 features likely configure the chronological age.

Furthermore, the data we use embed the chronological age, which is in turn used as discriminative information for external age groups. That is, it also partly explains the classification scores by showing that the two extreme age groups (age groups 1 and 6) are positioned at the two ends of the subspace and are thus easily discriminated, whereas the intermediate groups are in the middle with some overlapping, and are in turn difficult targets for discrimination.

### Finding the specific radiomorphometric features of the young and old

**Figure 5:**
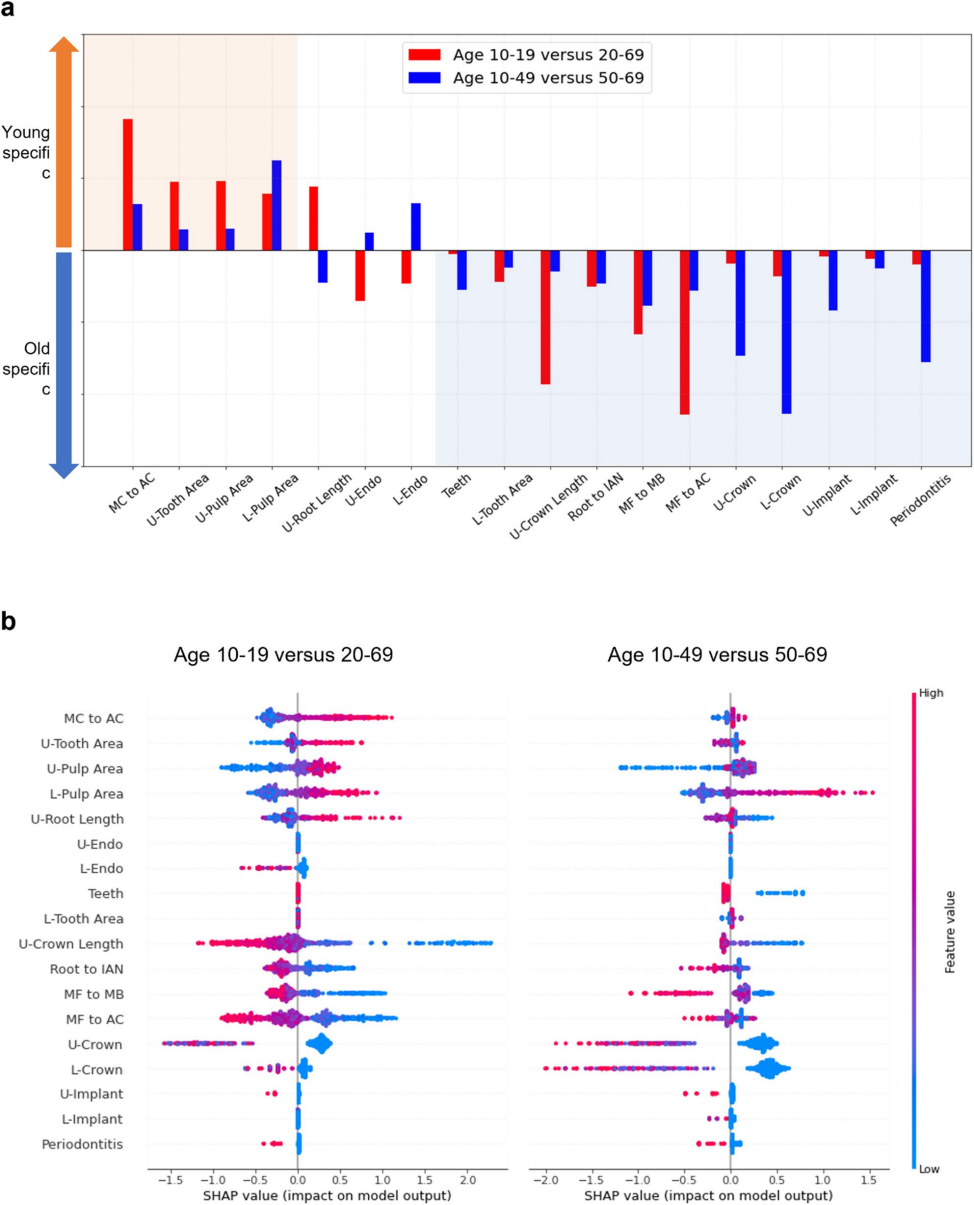
Feature analysis for LDA and XGBoost in young and old age group classifications (**a**) Learned weights of LDAs are shown as red bars (10–19 years versus 20–69 years) and old (10–49 years versus 50–69 years). The orange shaded area shows the features identified as young-specific: MC to AC, U-Tooth Area, U-Pulp Area, and L-Pulp Area. The light blue shaded area displays the old-specific features: Teeth, L-Tooth Area, U-Crown Length, Root to IAN, MF to MB, MF to AC, U-Crown, L-Crown, U-Implant, L-Implant, and Periodontitis. (**b**) SHAP values obtained from XGBoost in the classification of young and old age groups. Most of the young and old-specific features of XGBoost showed similar significance and age-related relationships to those of linear models.

To investigate significant features for age prediction, learned LDA feature weights were analyzed (Fig. 5a) and SHAP values were extracted from the trained XGBoost in young (10–19 years versus 20–69 years) and old (10–49 years versus 50–69 years) group classifications (Fig. 5b). Red and blue bars in Fig. 5a represent the LDA weights learned from young and old group classifications, respectively. Here, young-specific features show positive signs, and old-specific features are those showing negative signs. The weights from the old group are reversed for young- and old-specific features to achieve the same signs. The MC to AC, U-Tooth Area, U-Pulp Area, and L-Pulp area are shown as young-specific features (orange shaded area in Fig. 5). The L-Pulp area contributed to classifying ages of both 10–19 years and 10–49 years, whereas the other three features contributed specifically to classifying ages 10–19 years. That is, the higher the pulp area values of the maxillary and mandibular first molars, the more specific the features for younger ages (10–19 years). By contrast, the following 11 features were identified as old-specific features (blue shaded area): Teeth, L-Tooth Area, U-Crown Length, Root to IAN, MF to MB, MF to AC, U-Crown, L-Crown, U-Implant, L-Implant, and Periodontitis. Whereas U-Crown Length, MF to MB, and MF to AC were weighted more for the wider ages 20–69 years, features related to tooth damage or loss, i.e., Teeth, L-Crown, U-Crown, L-Implant, U-Implant, and Periodontitis, are reasonably weighed more for older ages of 50–69 years. The positive correlation between age and the increase in the total number of teeth in old ages may occur because the concept of “teeth” in our study does not include only natural teeth. Because the numbers of dental prostheses, including dental crowns and bridges, and dental implants were included, their number increased with age. In the case of the XGBoost, feature importance represented as SHAP values (b) shows similar significance to the linear case. First, higher values in MC to AC, U-Pulp Area, and L-Pulp area were also related to the young ages, as the LDA weights. Furthermore, L-Pulp was related to the ages 10–49 years, whereas the significance of MC to AC, U-Pulp Area was far higher in younger ages 10–19 years. On the other hand, most of the old specific features selected in the LDA case were also positive relationship with age, except for the number of teeth.

After specifying specific features of both the young and old, their predictive power using a simple linear regression was obtained (Table 4). The feature weight and statistical significance of individual features were tested to investigate the importance of each feature in the model. All features were weighted in the same directions as the weights learned from the LDA classifiers. All significant features from the LDA are also statistically significant for composing the linear model, except for L_Implant. The absolute value of the weight (− 4.265) of MC to AC was the highest, followed by L-Pulp Area (− 2.972), U-Tooth Area (− 2.324), and U-Pulp Area (− 1.886). That is, the decrease in age was linearly related to the increase in MC to AC, L-Pulp Area, U-Tooth Area, and U-Pulp Area. The fold mean NMSE of 0.496 $\pm$ 0.011, which is approximately 0.5, indicates that almost half of the lower MSE than that of the mean age was achieved by the linear model based on the selected features. U-Root Length, U-Endo, and L-Endo, which had inconsistent or insignificant effects on ages in LDA and XGBoost analysis were not included in the linear regression model.

**Table 4:** Result of linear regression using selected age-specific features.

	Radiomorphometric features	Weights	Standard errors	95% CI	p-value
Young-specific features (4)	MC to AC	− 4.265	0.864	− 5.958 to − 2.572	< 0.01**
	U-Tooth area	− 2.324	0.796	− 3.885 to − 0.764	< 0.01**
	U-Pulp area	− 1.886	0.505	− 2.876 to − 0.896	< 0.01**
	L-Pulp area	− 2.972	0.505	− 3.962 to − 1.982	< 0.01**
Old-specific features (11)	Teeth	1.394	0.455	0.502 to 2.286	< 0.01**
	L-Tooth area	2.213	0.856	0.535 to 3.891	< 0.01**
	U-Crown length	3.567	0.532	2.525 to 4.610	< 0.01**
	Root to IAN	3.315	0.733	1.878 to 4.752	< 0.01**
	MF to MB	2.856	0.546	1.785 to 3.926	< 0.01**
	MF to AC	2.261	0.572	1.140 to 3.383	< 0.01**
	U-Crown	3.440	0.572	2.319 to 4.561	< 0.01**
	L-Crown	4.170	0.609	2.976 to 5.364	< 0.01**
	U-Implant	1.027	0.500	0.047 to 2.007	< 0.05*
	L-Implant	0.688	0.507	− 0.306 to 1.682	0.175
	Periodontitis	1.528	0.506	0.536 to 2.520	< 0.01**

The feature weights and their statistical significance are presented. P-values < 0.05 (*) and < 0.01 (**) are recognized as statistically significant. *CI* confidence interval.

## Discussion

Our results were based on five machine learning algorithms (LDA, LR, SVM, MLP, and XGBoost) using 18 radiomorphometric parameters of PRs: (1) The parameters that primarily contributed to age group estimation differed by age. (2) The prediction accuracy of the machine learning model did not differ according to the age group, revealing higher values in the young and old age groups than in the adult age group. In addition, the prediction accuracy between the linear and nonlinear models was not statistically significantly different. (3) The prediction accuracy of the machine learning algorithm exceeded that of the existing age estimation methods and was acceptable. In particular, the model with LDA classified young (10–19 years) and elderly (50–69 years) age-specified information, and age-related feature weights were obtained. To the best of our knowledge, this is the first study on dental age-group estimation using multiple machine learning algorithms with various radiomorphometric parameters in the PRs.

Automatic age group estimation achieved an excellent prediction accuracy in both the young and old groups, and an acceptable level the middle-age group. A machine learning-based age estimation study using PR is meaningful, because PR is a basic radiography in the dental and forensic field, and the data derived from PR have reliability^20^. In the present study, the AUC values in the young group were 0.8576–0.8730, and those in the old group were 0.8733–0.8998. Pinchi et al. reported an AUC value of 0.87 when age estimation was applied using the Demirijan method with PRs for adolescents aged 11–16 years^21^. However, the age estimation method based on tooth development and maturation is definitely limited within an applicable age, and is used only in those aged 3.5–16.9 years, and it is impossible to estimate the results from those 17 years of age or older^22^.

By contrast, this study has relative strengths because it can be applied to individuals in their teens and 60 s. When revealing the identity and age of a dead or living person, an age estimation method applicable to all ages will be required^23^. In addition, if identification of a large number of disaster victims is required, an accurate and automated age estimation algorithm might be needed^16,24^. Thus, our results could provide a valuable tool for age estimation in refugee administration or forensic science. To ensure the validity of the machine learning algorithm, the data must be homogeneous and sufficient^25^. However, our training dataset was biased towards younger ages, so the predictions could also be biased towards younger ages. In our previous study, based on the first molar image and a CNN, the AUC values of the two extreme age groups were higher than those of the middle-aged group^16^. By further increasing the input data, we need to apply a machine learning model and find a way to increase the prediction accuracy in the middle-aged group. In the multi-label classifications, the accuracy was approximately 0.66 for three age groups and 0.47 for six age groups. Whether three or six group case, the predictions were biased to young ages. This would be explained by the limitation of the data set, where approximately 58% of the samples were younger than 29 years old (37% for 10–19 years and 21% for 20–29 years). For the accurate evaluation for machine learning algorithms in the multi-label setting using given features, more data from older ages seems to be necessary.

Given the forensic data, it is important to estimate age and sex. Recently, by applying the wide ResNet model to facial images, age and gender have been excellently distinguished^26^. In the traditional non-automatic method, the age estimation formula or trend has been obtained separately because males and females have different progression of tooth and bone development and aging processes^11,27,28^. However, using data augmentation and a CNN, age and gender can be automatically distinguished beyond that achievable by a human observer^26,29,30^. Technological innovation in artificial intelligence has led to an era in which it is no longer necessary to divide the dataset by gender, and it is possible to easily estimate the age and gender of unidentified persons. According to Wilczek et al., when root pulp in the mandibular third molars in PRs was used to estimate the age of 18 year-olds using a traditional method, the AUC value was 0.930 for men and 0.829 for women^31^. In the present study, the AUC value of age group estimation in males was significantly higher than that in females when applying XGBoost. However, there was no gender difference with other machine learning models, and the age group prediction accuracy was more than acceptable.

To understand the relationship between chronological age and age-specific features, we first analyzed the learned the weights of two LDAs in the classification of young and old groups. Additional analysis was performed using SHAP values from XGBoost models. Based on the results, the pulp area of the maxillary or mandibular first molar is a major biomarker for age estimation. The pulp area of teeth decreases with age owing to secondary dentin deposition, tooth mineralization, and pulp atrophy, and this relationship has been found in canines and molars^16,32,33^, which are teeth that remain in the human oral cavity for a relatively long period of time. Conversely, a large pulp area is useful for discriminating young ages. In the elderly aged 50–69 years, the presence of periodontitis and features related to tooth damage or loss were more associated with an increase in age. Periodontitis can occur in any age group, its prevalence increases with age, reaching 25.8% in people over 65 years of age^34^. It has also been reported that the number of missing teeth, endodontic teeth, full veneered crowns, and implant prostheses increase with age^11^. Because tooth damage, tooth loss, and the presence of periodontitis are influenced by the environment and genetics, it may show a complex trend rather than a unidirectional increase/decrease trend in middle-aged groups, and thus more data and research based on developed algorithms are needed.

Crucial structures in the mandible, such as the MC and MF, were comprehensively examined in the age group estimation. The increase in MC to AC was related to young age, and the decrease in MF to AC was related to old age. In the elderly, both MC and MF gradually approach the alveolar bone crest, based on the degree of bone resorption and tooth loss^35^. When considering MF, the location and size of the MF vary with gender and race^36^. However, it is not clear how MC and MF work among middle-aged groups. In particular, this trend has not been investigated in individuals who do not suffer from periodontitis and have good oral health without significant tooth loss or tooth damage. Machine learning algorithms have a black-box phenomenon. According to Rudin et al., we should stop explaining black box machine learning models for high-stake decisions and use interpretable models instead^37^. That is, instead of completely leaving the feature-extraction step to the machine learning model as a black box, it is advisable to manually extract radiographic identification landmarks and use them to enhance the model performance, because they are indications of age^26,38,39^. We applied a machine learning algorithm by selecting 18 features that may be related to changes in age and investigated their extent; however, more research is required to confirm that a research direction of building a model with the selected features is correct.

The application of artificial intelligence in the field of forensics is an unstoppable major trend. We first conducted binary classifications for the age group using various machine learning algorithms and analyzed the learned feature weights for all age groups. The result of FDA revealed that the data configuration showing age information indeed embedded and it can be captured in a 2D space. Finally, we could select 14 features that are closely related to ages. However, the present study has a few limitations: First, because we have an insufficient number of samples, there might be a risk of complex model such as XGBoost was overfitted and thus were not fully-functioned^40^. Additional classification with larger sample size is needed to determine whether the prediction performance of XGBoost will increase. Second, the study population was biased toward younger ages of under 30 y because they were the main visitors during the data collection period. Because there was no difference in performance between XGBoost and the linear algorithms in both binary classifications and multi-label categorizations, additional tests are needed to determine whether the performance of XGBoost in handling complex data will increase if the dimensions of the data are increased and will be improved if the amount of data are balanced and supplemented. Finally, although we investigated unexpected weights in two features by using prior knowledge and the characteristics of a single algorithm, there might be unknown interactions among the other features. To analyze the relationship with age more precisely, feature interaction should be considered and investigated.

## Data Availability

The datasets generated and/or analyzed during this study are available from the corresponding author upon reasonable request. Because patient consent is required for data disclosure, we may disclose data conditionally through internal discussions and the Institutional Review Board (IRB) of the Kyung Hee University Dental Hospital.

## References

[1] Schmeling A, Geserick G, Reisinger W, Olze A (2007). Age estimation. Forensic Sci. Int..

[2] Manzoor Mughal A, Hassan N, Ahmed A (2014). Bone age assessment methods: A critical review. Pak. J. Med. Sci..

[3] Verma M, Verma N, Sharma R, Sharma A (2019). Dental age estimation methods in adult dentitions: An overview. J. Forensic Dent. Sci..

[4] Lacruz RS, Habelitz S, Wright JT, Paine ML (2017). Dental enamel formation and implications for oral health and disease. Physiol. Rev..

[5] Demirjian A, Goldstein H, Tanner JM (1973). A new system of dental age assessment. Hum. Biol..

[6] Demirjian A, Goldstein H (1976). New systems for dental maturity based on seven and four teeth. Ann. Hum. Biol..

[7] Willems G, Van Olmen A, Spiessens B, Carels C (2001). Dental age estimation in Belgian children: Demirjian's technique revisited. J. Forensic Sci..

[8] Metzger Z, Buchner A, Gorsky M (1980). Gustafson's method for age determination from teeth—A modification for the use of dentists in identification teams. J. Forensic Sci..

[9] Warreth A, Abuhijleh E, Almaghribi MA, Mahwal G, Ashawish A (2020). Tooth surface loss: A review of literature. Saudi Dent. J..

[10] Sue M, Oda T, Sasaki Y, Ogura I (2018). Age-related changes in the pulp chamber of maxillary and mandibular molars on cone-beam computed tomography images. Oral. Radiol..

[11] Lee YH, Auh QS, Chun YH, An JS (2021). Age-related radiomorphometric changes on panoramic radiographs. Clin. Exp. Dent. Res..

[12] Eklund SA (2010). Trends in dental treatment, 1992 to 2007. J. Am. Dent. Assoc..

[13] Nazir MA (2017). Prevalence of periodontal disease, its association with systemic diseases and prevention. Int. J. Health Sci. (Qassim).

[14] Othmani A, Taleb AR, Abdelkawy H, Hadid A (2020). Age estimation from faces using deep learning: A comparative analysis. Comput. Vis. Image Underst..

[15] Sarker IH (2021). Machine learning: Algorithms, real-world applications and research directions. SN Comput. Sci..

[16] Kim S, Lee YH, Noh YK, Park FC, Auh QS (2021). Age-group determination of living individuals using first molar images based on artificial intelligence. Sci. Rep..

[17] Shen S (2021). Machine learning assisted Cameriere method for dental age estimation. BMC Oral Health.

[18] Metz CE (1978). Basic principles of ROC analysis. Semin. Nucl. Med..

[19] Onel M, Kieslich CA, Pistikopoulos EN (2019). A nonlinear support vector machine-based feature selection approach for fault detection and diagnosis: Application to the Tennessee eastman process. AIChE J..

[20] Makkad RS (2013). Reliability of panoramic radiography in chronological age estimation. J. Forensic Dent. Sci..

[21] Pinchi V (2016). Comparison of the diagnostic accuracy, sensitivity and specificity of four odontological methods for age evaluation in Italian children at the age threshold of 14 years using ROC curves. Med. Sci. Law.

[22] Yan J (2013). Assessment of dental age of children aged 3.5 to 16.9 years using Demirjian's method: A meta-analysis based on 26 studies. PLoS One.

[23] Alkass K (2010). Age estimation in forensic sciences: Application of combined aspartic acid racemization and radiocarbon analysis. Mol. Cell Proteomics.

[24] Heinrich A, Güttler FV, Schenkl S, Wagner R, Teichgräber UKM (2020). Automatic human identification based on dental X-ray radiographs using computer vision. Sci. Rep..

[25] Janiesch C, Zschech P, Heinrich K (2021). Machine learning and deep learning. Electron. Mark..

[26] Huynh HT, Nguyen H (2020). Joint age estimation and gender classification of Asian faces using wide ResNet. SN Comput. Sci..

[27] do Nascimento LG (2020). Age estimation in north east Brazilians by measurement of open apices. J. Forensic Odontostomatol..

[28] Verochana K, Prapayasatok S, Janhom A, Mahasantipiya PM, Korwanich N (2016). Accuracy of an equation for estimating age from mandibular third molar development in a Thai population. Imaging Sci. Dent..

[29] Liu X, Zou Y, Kuang H, Ma X (2020). Face image age estimation based on data augmentation and lightweight convolutional neural network. Symmetry.

[30] Tursunov A, Mustaqeem Choeh JY, Kwon S (2021). Age and gender recognition using a convolutional neural network with a specially designed multi-attention module through speech spectrograms. Sensors (Basel)..

[31] Akkaya N, Yılancı HÖ, Boyacıoğlu H, Göksülük D, Özkan G (2019). Accuracy of the use of radiographic visibility of root pulp in the mandibular third molar as a maturity marker at age thresholds of 18 and 21. Int. J. Legal Med..

[32] Juneja M, Devi YBK, Rakesh N, Juneja S (2014). Age estimation using pulp/tooth area ratio in maxillary canines—A digital image analysis. J. Forensic Dent. Sci..

[33] Ge ZP, Ma RH, Li G, Zhang JZ, Ma XC (2015). Age estimation based on pulp chamber volume of first molars from cone-beam computed tomography images. Forensic Sci. Int..

[34] Eke PI (2016). Periodontitis prevalence in adults ≥ 65 years of age, in the USA. Periodontol 2000.

[35] Bhardwaj D, Kumar JS, Mohan V (2014). Radiographic evaluation of mandible to predict the gender and age. J. Clin. Diagn. Res..

[36] Apinhasmit W, Methathrathip D, Chompoopong S, Sangvichien S (2006). Mental foramen in Thais: An anatomical variation related to gender and side. Surg. Radiol. Anat..

[37] Rudin C (2019). Stop explaining black box machine learning models for high stakes decisions and use interpretable models instead. Nat. Mach. Intell..

[38] Ching T (2018). Opportunities and obstacles for deep learning in biology and medicine. J. R. Soc. Interface.

[39] Lundervold AS, Lundervold A (2019). An overview of deep learning in medical imaging focusing on MRI. Z. Med. Phys..

[40] Schmidt J, Marques MRG, Botti S, Marques MAL (2019). Recent advances and applications of machine learning in solid-state materials science. NPJ Comput. Mater..

